# A dedicated preventive protocol sustainably avoids spinal cord ischemia after endovascular aortic repair

**DOI:** 10.3389/fcvm.2024.1440674

**Published:** 2024-08-01

**Authors:** Lina Rosvall, Angelos Karelis, Björn Sonesson, Nuno V. Dias

**Affiliations:** ^1^ Department of Clinical Sciences Malmö, Faculty of Medicine, Lund University, Malmö, Sweden; ^2^ Vascular Center, Department of Thoracic Surgery and Vascular Diseases, Skåne University Hospital, Malmö, Sweden

**Keywords:** complex endovascular aortic aneurysms, spinal cord ischemia, endovascular aortic repair, spinal cord ischemia prevention, endovascular complications, spinal cord ischemia risk factors

## Abstract

**Objective:**

To analyze the incidence of spinal cord ischemia (SCI) after complex endovascular aortic repair (EVAR) after the introduction of a dedicated SCI preventive protocol.

**Methods:**

Retrospective review of all consecutive patients undergoing complex EVAR with branched (BEVAR) and/or fenestrated grafts (FEVAR) during a 6-year period starting January 1st, 2015. The preventive protocol consisted of staging extensive aortic repairs, maintaining a mean arterial pressure (MAP) >80 mm Hg, Hb level >110 g/L, early lower limb reperfusion and neurological control per hour during the post-operative stay in the intensive care unit (36–72 h). Prophylactic cerebrospinal fluid drainage (CSFD) was used selectively. Pre- intra-, and 30-day postoperative clinical data and imaging were collected. Primary end point was the development of perioperative SCI. Secondary outcome included technical and clinical success.

**Results:**

Complex EVAR was performed in 205 patients (167 males, 72 (67–75) years, 182 (88.8%) elective) with juxtarenal aneurysms (JRA, 155 patients) or thoracoabdominal aortic aneurysms (TAAA). SCI occurred after JRA repair in two patients (1.3%, both ruptures) and after TAAA repair in three (6.0%, one rupture) (*p* = 0.06), all within 9 h postoperatively. There was symptom regression in three cases (one partial, two complete), resulting in a persistent SCI level of 0.6% and 4.0% for JRA and TAAA, respectively. Only one patient with persistent SCI could be discharged from the hospital alive. Patients developing SCI were more commonly female (*n* = 3, *p* = .016), presented with rupture (*n* = 3, *p* < .001), had preoperative renal insufficiency (*n* = 5, *p* < .001) and had lower minimal MAP (*p* = .015). No regression analysis was done due to the limited number of SCI events in relation to the study population size. Primary technical success was achieved in 162 patients (83.5%) and clinical success in 153 patients (75.4%), without any differences between the groups.

**Conclusions:**

The incidence of persistent SCI after complex EVAR is low with the use of a dedicated SCI preventive protocol allowing the early diagnosis. Females, patients with ruptured aneurysms and preoperative renal insufficiency are at higher risk. Further studies are needed to customize the protocols particularly in those high-risk patients.

## 1 Introduction

Complex endovascular aortic repair (EVAR) has become the predominant treatment of juxtarenal aneurysms (JRA) and thoracoabdominal aortic aneurysms (TAAA) using fenestrated (FEVAR) and/or branched (BEVAR) stentgrafts during the last decade (1, 2). However, spinal cord ischemia (SCI) remains one of the most feared complications with incidences ranging from 0% to 35% (3, 4). The symptoms of SCI vary from mild deficits to complete paraplegia and are commonly presented within 72 h postoperative (5, 6). The extent of the disease has been the most important risk factor for SCI development, as already found in cases of open repair. However, several other risk factors have been identified for EVAR, mostly assumed to be related to the perfusion of the spinal cord and the development of a collateral network. This includes the simultaneous coverage of several segments of aortic collateral branches; as well as keeping high haemoglogin levels, mean arterial pressure (MAP) and oxygenation of the blood. Several preventive strategies have been suggested based on these identified pre-, intra-, and postoperative risk factors. These include staging of repairs involving long aortic coverage; revascularization of collateral beds such as the left subclavian artery (LSA) and hypogastric arteries; early intraoperative reperfusion of the lower limbs; usage of cerebrospinal fluid drainage (CSFD); optimized blood oxygenation, MAP and glucose levels; embolization of intercostal and lumbar arteries; administrating of steroids and naloxone; and frequent postoperative neurological controls to allow an early identification of deficits (3, 7–12). The extent of the aortic repair has been associated with SCI incidence after EVAR, but little has been studied on the results by preoperative anatomic extent in relation to the emergency setting of the repair and the effects of a dedicated protocol including the majority of the measures above. In an initial report we saw a significant reduction of SCI with the progressive introduction of some of the measures mentioned above (13). The aim of this study is to analyze the incidence of SCI in patients that were treated with complex EVAR in the same tertiary center after the full implementation of this protocol. Furthermore, risk factors for developing SCI when the protocol is systematically used will also be assessed.

## 2 Methods

### 2.1 Patient population

The study followed the STrengthening the Reporting of OBservational Studies in Epidemiology (STROBE) statement. All consecutive patients who underwent F/BEVAR of complex aortic aneurysms after the complete introduction of a dedicated SCI preventive protocol at a tertiary university center during a 6-year period starting January 1st 2015 were identified. This period was chosen since it followed the initial assessment of the progressive introduction of the protocol. Patients' charts and imaging were retrospectively reviewed according to a predefined protocol, based on the reporting standards for EVAR of aneurysm in the abdominal, thoracic, and engaging renovisceral segments (14–16). Inclusion in the study required that all patent renovisceral arteries were incorporated in the repair and that the patients were monitored according to the preventive protocol. In elective TAAA requiring extensive thoracic aortic coverage (TAAA Crawford I–III), the operation was staged in two sessions, most commonly with an initial thoracic EVAR followed by the BEVAR or FEVAR performed later. TAAA repairs performed acutely were done in a single stage independently of the extent of the aneurysm. No intercostal and/or lumbar artery selective embolization were done as part of a staging strategy. Previous aortic surgery did not include initial step of planned stage procedures. Patients that died during the perioperative period without the opportunity to do a wake-up test for neurological assessment or that died in-between stages of the repair (before the exclusion of the aneurysm) were not included in the study.

### 2.2 Imaging

Preoperative thin slice contrast-enhanced computer tomography angiography (CTA) that was used for the planning of the repair was reviewed. The aneurysms were classified as JRA or TAAA, the latter being further categorized according to the modified Crawford classification (16). The maximal aneurysm diameter was measured in axial imaging, using the perpendicular to the largest diameter to avoid overestimation due to vessel tortuosity. The patency of LSA, visceral, renal, and hypogastric arteries were classified as either being occluded, significantly stenosed (≥50%) or patent (0%–49%). The sealing zones were noted to define the extent of aortic coverage, as proposed by the reporting standards (15, 16). The three-phased CTA done one month postoperatively was reviewed to identify any aneurysm progression, stentgraft migration, endoleak (EL) or altered graft patency.

### 2.3 Endovascular procedure

All procedures were carried out under general anesthesia in a hybrid operation room under fusion imaging guidance, with use of both iodine and carbon dioxide as contrast agents by the same experienced surgeons. Percutaneous arterial access was obtained from both femoral arteries and the brachial or axillary artery whenever necessary. Stentgrafts with branches, fenestrations, or a combination of both (Cook Inc, Bloomington, IN, USA) were used. The grafts were either custom-made for the patient or off-the-shelf if suitable, intending to preserve patent renovisceral arteries with a diameter ≥3 mm. Off-the-shelf devices consisted of either the P- or T-branch (Cook Inc, Bloomington, IN, USA) (17, 18). *In situ* fenestrated or physician modified grafts for the reno-visceral segment were not included. Proximal thoracic and distal abdominal grafts were used whenever needed to extend the F-BEVAR component to appropriate sealing zones proximally and distally. This included the use of fenestrations, branches and/or chimneys to the subclavian and hypogastric arteries if needed to avoid their embolization. Early lower limb perfusion was achieved in branched repairs by expedite deployment of all the aortic grafts followed by downsizing the femoral introducer with retraction of the pre-closure sutures to 7–12 Fr depending on the needs for subsequent accessing the branches. In fenestrated repairs, preloaded devices for the renal arteries were routinely used permitting the use of a 7–12 Fr sheath into the contralateral access site during most procedural steps. Motor-evoked potentials (MEP) monitoring was used in selected cases for neurological monitoring during surgery. A final intraoperative completion angiography was performed to confirm technical success, with Cone beam CT included as part of the completion control in all cases. Technical success was defined according to the reporting standards and included: successful access to the arterial system; successful delivery and deployment of stent graft; successful side branch catheterization; absence of type I and III EL that extends beyond 30 days; and patency of all stent graft components (16).

### 2.4 Postoperative care and complications

Patients with JRA repair were monitored overnight in the postoperative recovery unit and discharged to the vascular surgery ward the next morning. Patients with TAAA repair, or in need of intensive care monitoring for other reasons, were monitored in ICU for 36–72 h postoperatively. All patients had invasive arterial line monitoring with medical support to achieve the goals included in the protocol described below. Clinical success was defined according to the reporting standards (16). In summary, it requires technical success, as described above, in addition to the absence of important clinical disabilities including death; surgical conversion, aneurysm rupture or graft infection; and permanent SCI, stroke or dialysis. In addition to these events, all major complications that occurred within the first 30 days postoperative, or during the initial hospitalization, were noted. Major complications were defined as events that prolonged the hospital stay >24 h, entailed medical and/or surgical interventions, or resulted in permanent disabilities. The nature of the events was classified as being related to organ function, device, or procedure. Deaths occurring within 30 days following the operation or during hospitalization were categorized as EVAR-related.

### 2.5 Spinal cord ischemia and preventive protocol

The dedicated SCI preventive protocol applied after complete exclusion of the aneurysm included staging extensive aortic repairs and expedite removal or downsizing of the large sheaths to allow the earliest possible reperfusion of the lower limb. After the exclusion of the aneurysm, measures were taken to maintain a medically driven MAP >80 mm Hg, Hb > 110 g/L and maximal blood oxygenation. Postoperatively, patients were admitted to ICU or recovery unit with neurological controls every hour to allow early detection of new neurological deficits. This was done overnight for JRA and for 36–72 h in TAAA. Prophylactic CSFD was used routinely in the initial part of this study period in patients undergoing the last stage of TAAA repairs extent I–III. For the first stage of these repairs and for all others, CSFD was placed only upon the development of symptoms. Whenever used, prophylactic drains were inserted immediately before the induction of anesthesia and used for 36 h postoperatively. CSFD placed for symptom development were left in place for at least 72 h. CSFD was set passively initially at 10 mm Hg and in case of symptom development and/or non-regression, it was decreased to 5 mm Hg to maximize the output up to 15 ml/h. The SCI diagnosis was set by an independent neurologist and the neurological function was graded at symptom development and at discharge according to the reporting standards, to account for any progress and/or regress (16). Any patients with neurological deficits underwent a CT or MR of the brain and spinal cord in addition to the neurological examination.

### 2.6 Data management and statistical analysis

The authors had full access to and take full responsibility for the integrity of the data. This article is part of a larger study which has been granted ethical approval (Dnr: 2014/732). Categorial data were presented as absolute numbers and percentage (%), while continuous were expressed as median with interquartile range (IQR). To test significance between groups, Chi-square test was used for categorial data and Mann-Whitney *U*-test for continuous. *P*-values were rounded down to third decimal place and considered significant whenever *p *< .05. Statistical analysis was performed with SPSS, version 26.0 (IBM Corp., Armonk, N.Y.). The relative low number of events limited the possibility to perform any reliable multivariate regression at this stage.

## 3 Results

### 3.1 Patient characteristics and anatomy

A total of 232 patients underwent EVAR of complex aortic aneurysm with F/BEVAR. Of these, 205 patients (167 males (81.5%), median age of 72 (67–75) years) were eligible for the final analysis (Supplementary Figure 1). The aneurysms were JRA in 155 cases (75.6%) and TAAA in 50 (24.4%). Of the TAAA, 13 (26.0%) were Crawford type I, ten (20.0%) type II, ten (20.0%) type III, ten (20.0%) type IV and seven (14.0%) type V. Most of the patients were asymptomatic, while 14 (6.8%) were symptomatic and ten (4.9%) presented with rupture (seven JRA and three TAAA). There were no major differences in patient characteristics between patients with JRA and TAAA, except for a higher body mass index (BMI) in the former (*p *= .035), and a higher prevalence of cerebrovascular incidents (*p* = .001) and previous aortic surgery (*p* < .001) in the latter. Patient and aneurysm characteristics are given in Tables 1, 2. The extent of the aortic coverage is depicted in Supplementary Figure 2.

**Table 1:** Patient characteristics and comorbidities.

Patient characteristics	Total	Juxtarenal	TAAA	*P*-value
	(*n* = 205)	(*n* = 155)	(*n* = 50)	
Male gender	167 (81.5)	129 (83.2)	38 (76.0)	.253
Age, years	72 (67–75)	72 (67–75)	72 (67–75)	.499
Smoking				
Current	72 (35.1)	52 (33.5)	20 (40)	.406
Previous	104 (50.7)	83 (53.5)	21 (42)	.156
BMI, kg/m^2^	26.3 (23–29)	26.8 (24–29)	25.0 (22–27)	.035
ASA ≥ 3	184 (91.5)	136 (89.5)	48 (98.0)	.063
Myocardial infarction	45 (22.0)	38 (24.5)	7 (14.0)	.118
Atrial fibrillation	24 (11.7)	17 (11.0)	7 (14.0)	.562
Congestive heart failure	18 (8.8)	14 (9.0)	4 (8.0)	.823
Cerebrovascular disease	34 (16.6)	18 (11.6)	16 (32.0)	.001
COPD	52 (25.4)	37 (23.9)	15 (30.0)	.386
eGFR ≤ 60 ml/min/1.73 m^2^	54 (26.3)	36 (23.2)	18 (36.0)	.075
Peripheral artery disease	89 (43.4)	62 (40.0)	27 (54.0)	.082
ABI < 0.9	44 (21.5)	29 (18.7)	15 (30.0)	
ABI > 1.1	35 (17.1)	26 (16.8)	9 (18.0)	
Previous intervention	10 (4.9)	7 (4.5)	3 (6.0)	
Hypertension	148 (72.2)	108 (69.7)	40 (80.0)	.157
Diabetes	31 (15.3)	22 (14.2)	9 (18.8)	.443
DVT or pulmonary embolism	8 (3.9)	6 (3.9)	2 (4.0)	.967
Connective tissue disease	1 (0.5)	0 (0)	1 (2.0)	.079
Previous aortic surgery				<.001
Open ascending and/or arch	5 (2.4)	0 (0)	5 (10.0)	
Other^a^	49 (23.9)	32 (20.6)	17 (34.0)	

Categorial variables are presented as number (%). Continuous variables are presented as median (IQR).

^a^ Including open or endovascular procedure in abdominal or thoracic aorta.

**Table 2:** Aneurysm characteristics.

Aneurysm characteristics	Total	Juxtarenal	TAAA	*P*-value
	(*n* = 205)	(*n* = 155)	(*n* = 50)	
Clinical presentation				0.59
Asymptomatic	181 (88.3)	141 (91.0)	40 (80.0)	
Symptomatic	14 (6.8)	7 (4.5)	7 (14.0)	
Rupture	10 (4.9)	7 (4.5)	3 (6.0)	
Crawford type				
Type I			13 (26.0)	
Type II			10 (20.0)	
Type III			10 (20.0)	
Type IV			10 (20.0)	
Type V			7 (14.0)	
Maximal diameter, mm	60.0 (56–67)	59.0 (55–65)	62.5 (60–71)	.001
Accessory renals				.369
1	56 (27.3)	37 (23.9)	19 (38.0)	
≥2	7 (3.4)	6 (3.9)	1 (2.0)	
Other anatomic variations	7 (3.4)	6 (3.9)	1 (2.0)	.526
Pre-operative occlusion/stenosis				
LSA	2 (1.0)	1 (0.6)	1 (2.0)	.397
Celiac artery	21 (10.2)	9 (5.8)	12 (24.0)	<.001
SMA	9 (4.4)	6 (3.9)	3 (6.0)	.523
Renal arteries	20 (9.8)	12 (7.7)	8 (16.0)	.013
Hypogastric arteries				.263
Unilateral	20 (9.8)	18 (11.6)	2 (4.0)	
Bilateral	6 (2.9)	4 (2.6)	2 (4.0)	

LSA, left subclavian artery; SMA, superior mesenteric artery.

Categorial variables are presented as number (%). Continuous variables are presented as median (IQR).

### 3.2 Intraoperative details and technical success

Patient received grafts bridging a median of 4 (4–4) renovisceral arteries. In the JRA population, 146 patients (94.2%) received FEVARs while seven patients received BEVARs, of which five were treated subacutely or acutely with off-the-shelf stentgrafts. The remaining two patients with JRA received grafts with both branches and fenestrations. Half of the patients with TAAA received BEVARs (24 patients, 48.0%), while 17 (34.0%) had FEVARs implanted. The remaining nine patients (18.0%) had a combination of both. The median proximal sealing zone was lower in JRA than for TAAA (zone 5 (5–5) vs. 4 (3–5), respectively, *p* < .001). Consequently, and naturally, the extent of aortic coverage was more extensive for TAAA patients than for the ones undergoing repair of a JRA (Supplementary Figure 2). Embolization of hypogastric arteries was necessary in six patients with JRA (all unilateral and all elective). Revascularization of LSA was done in six TAAA patients (12.0%), including two LSA chimney; two carotid-to-LSA-bypass; one LSA fenestration; and one combination of carotid chimney and LSA fenestration. Eighteen patients with TAAA (36.0%) underwent repair in two stages, while all other had single stage repairs. The operation time and intraoperative bleeding were significantly greater in the TAAA group (*p* = .013 and *p* = .016, respectively). Primary technical success was achieved in 162 patients (83.5%) without differences between the groups. Secondary technical successes could be achieved in four patients after an additional procedure to correct an EL or target vessel instability. The most common cause of technical failure was a non-patent target vessel [15 patients (7.7%), 14 JRA of which one rupture and one symptomatic TAAA,] and a persistent EL type I or III [13 (6.7%), ten JRA of which one rupture, three asymptomatic TAAA]) on the 1-month CTA. Eleven patients did not undergo the 1-month CTA (9 deaths, 2 loss of follow-up); hence they were censored from the postoperative imaging analysis. Intraoperative details are given in Table 3.

**Table 3:** Intraoperative details and technical success.

Operation details	Total	Juxtarenal	TAAA	*P*-value
	(*n* = 205)	(*n* = 155)	(*n* = 50)	
Operation type				.030
Elective	182 (88.8)	142 (91.6)	40 (80.0)	.024
Subacute	18 (8.8)	9 (5.8)	9 (18.0)	.008
Acute	5 (2.4)	4 (2.6)	1 (2.0)	.789
Operation time, minutes	268 (208–368)	262 (204–364)	303 (246–400)	.013
Intra-operative bleeding, ml	400 (200–1,000)	400 (200–800)	500 (300–1,150)	.016
Stentgraft				
Off the shelf	24 (11.7)	10 (6.5)	14 (28.0)	<.001
Proximal attachment zone	5 (5–5)	5 (5–5)	4 (3–5)	<.001
Distal attachment zone	10 (10–11)	10 (10–11)	10 (9–11)	.001
Nr of target vessels	4 (4–4)	4 (4–4)	4 (4–4)	.092
Only fenestrations	163 (79.5)	146 (94.2)	17 (34.0)	<.001
Only branches	31 (15.1)	7 (4.5)	24 (48.0)	<.001
Both	11 (5.4)	2 (1.3)	9 (18.0)	<.001
Staged procedure	18 (8.8)	0 (0.0)	18 (36.0)	<.001
Adjunctive procedure				
Emb. accessory renals				.195
1	37 (18.0)	23 (14.8)	14 (28.0)	
≥2	5 (2.5)	4 (2.6)	1 (2.0)	
Emb. hypogastric arteries	6 (2.9)	6 (3.9)	0 (0)	.367
Revascularization LSA	6 (2.9)	0 (0.0)	6 (12.0)	<.001
Contrast				
Volume, ml	107 (80–145)	103 (80–138)	120 (89–160)	.079
Iodine dose, g	15 (11–21)	15 (11–20)	17 (12–23)	.081
Intra-operative CO2 contrast use	172 (84.3)	126 (81.8)	46 (92.0)	.085
Fluoroscopy time, minutes	88 (66–117)	87 (64–117)	89 (73–125)	.408
Dose area product, Gy·cm^2^	13.2 (9.4–19.4)	13.8 (9.7–19.3)	12.4 (8.9–25.2)	.675
Technical success	162 (83.5)	121 (81.2)	41 (91.1)	.292
Failure due to patency	15 (7.7)	14 (9.4)	1 (2.2)	
Failure due to EL type I/III	13 (6.7)	10 (6.7)	3 (6.7)	

Emb, embolization; LSA, left subclavian artery; EL, endoleakage.

Categorial variables are presented as number (%). Continuous variables are presented as median (IQR).

### 3.3 Postoperative care and clinical success

Patients with JRA were hospitalized postoperatively for 5 (3–8) days and TAAA for 7 (5–10) days (*p* = .008). Clinical success was achieved in 153 patients (75.4%), without difference between the groups. Seven of these were secondary successes, three after endovascular reintervention due to EL, symptomatic aortic dissection, and symptomatic occlusion of the celiac trunk, respectively. The remaining four received an open surgical procedure due to postoperative bleeding or compartment syndrome. Nine patients (4.4%) were labelled clinical failure due to death within 30 days or during hospitalization, after a median time of 18 (6–30) days. All nine suffered multiple postoperative complications. One of these had presented with a rupture whereas the remaining eight were asymptomatic preoperatively. Other causes of clinical failure were SCI, stroke, or dialysis (13 patients); graft infection, compartment, or rupture (9 patients); and aneurysm sac expansion, stent migration or graft kinking (2 patients). The remaining were technical failures. One-month CTA was not done adequately with dedicated protocol to assess the outcome in two of the alive patients at that time, hence they were censored from the imaging analysis. Major procedure-related adverse events occurred in 27 patients (13.2%), making it the most common postoperative complication without any differences between the two groups. Renal function decline was noted in 20 patients (9.8%), of which five (2.4%) had an intraoperative renal artery occlusion or embolization. Thirty-one patients (15.1%) had two or more major complications details of postoperative care and complications can be found in Table 4.

**Table 4:** Postoperative care, complications, and clinical success.

Post-operative care and complications	Total	Juxtarenal	TAAA	*P*-value
	(*n* = 205)	(*n* = 155)	(*n* = 50)	
Hours in ICU and recovery unit	19 (7–46)	14 (6–21)	48 (40–67)	<.001
Days until discharge	6 (4–8)	5 (3–8)	7 (5–10)	.008
Major complications				
SCI	5 (2.4)	2 (1.3)	3 (6.0)	.060
Stroke				
Major	4 (2.0)	2 (1.3)	2 (4.0)	.228
Minor	5 (2.4)	2 (1.3)	3 (6.0)	.060
Cardiac^a^	7 (3.4)	4 (2.6)	3 (6.0)	.247
Respiratory failure	10 (4.9)	8 (5.2)	2 (4.0)	.740
Renal function decline^b^	20 (9.8)	16 (10.4)	4 (8.0)	.622
Intraoperative renal occlusion^c^	5 (2.4)	5 (3.2)	0 (0.0)	
Bowel ischemia	6 (2.9)	4 (2.6)	2 (4.0)	.605
Device-related^d^	4 (2.0)	4 (2.6)	0 (0.0)	.251
Procedure-related^e^	27 (13.2)	22 (14.2)	5 (10.0)	.446
Time to first event, days	1 (1–2)	1 (1–2)	2 (1–3)	.075
≥2 major events	31 (15.1)	20 (12.9)	11 (22.0)	.118
Clinical success	153 (75.4)	114 (74.0)	39 (79.6)	.431
30-day mortality	9 (4.4)	5 (3.2)	4 (8.0)	.152
Days, median	18 (6–30)	16 (6–58)	22 (7–28)	.806

UVA, uppvakningsavdelningen; ICU, intensive care unit; SCI, spinal cord ischemia.

Categorial variables are presented as number (%). Continuous variables are presented as median (IQR).

^a^ Myocardial infarction, acute congestive heart failure, arrythmia.

^b^ Renal function decline resulting in >50% reduction in baseline eGFR or new-onset dialysis.

^c^ Number of patients with intraoperative renal occlusion among those with renal decline.

^d^ Graft or branch occlusion.

^e^ Dissection, pseudoaneurysm, distal thrombosis, compartment, bleeding from vessel included in procedure.

### 3.4 Preventive protocol and spinal cord ischemia

Information regarding SCI risk factors and SCI preventive protocol is presented in Table 5. CSFD was placed prophylactically before the repair in 27 cases (13.2%) and postoperatively upon the development of symptoms assumed initially to be SCI in five additional patients (2.4%). However, SCI was ultimately ruled out in four of these cases. The symptoms were later attributed to either psychiatric causes or another neurological event such as minor stroke. Two patients with TAAA type II (one connective tissue disease) receiving prophylactic CSFD (7.4%), developed symptomatic subdural hematomas, one of which remained completely paraplegic despite neurosurgical intervention. In one additional patient, blood was encountered in the CSFD although no bleeding was detected on the CT-scan. The CSFD was removed without any symptoms or complications.

**Table 5:** Spinal cord ischemia risk factors and preventive protocol.

SCI risk factors and preventive protocol	SCI within 30 days	No SCI	*P*-value
	*n* = 5	*n* = 200	
Age	74 (67–79)	72 (67–75)	.440
Male gender	2 (40.0)	165 (82.5)	.016
COPD	2 (40.0)	50 (25.0)	.446
eGFR < 60 ml/min/1.73 m^2^	5 (100.0)	49 (24.5)	<.001
Hypertension	5 (100.0)	143 (71.5)	.160
Presented with rupture	3 (60.0)	7 (3.5)	<.001
Location			.023
TAAA Crawford type I	2 (40.0)	11 (5.5)	
TAAA Crawford type III	1 (20.0)	9 (4.5)	
Juxtarenal	2 (40.0)	153 (76.5)	
Maximal aneurysm diameter	71 (53–85)	60 (56–67)	.217
Contrast volume	103 (78−162)	107 (81–145)	.099
Operation time	368 (230–688)	268 (208–365)	.174
Bleeding	500 (250–4,500)	400 (200–988)	.349
SCI preventive protocol			
Lowest MAP	60 (45–72)	73 (68–80)	.015
Lowest Hb	106 (82–112)	102 (93–112)	.888
Staged procedure	0 (0)	18 (9.0)	.482
MEP during surgery	0 (0.0)	22 (11.1)	.431
Prophylactic CSFD	4 (80.0)	23 (11.6)	<.001
Symptomatic CSFD	1 (20.0)	4 (2.0)	.010

TAAA, thoracoabdominal aortic aneurysm; SCI, spinal cord ischemia; MAP, mean arterial pressure; MEP, motor-evoked potentials; CSFD, cerebrospinal fluid drainage.

Categorial variables are presented as number (%). Continuous variables are presented as median (IQR).

**Figure 1:**
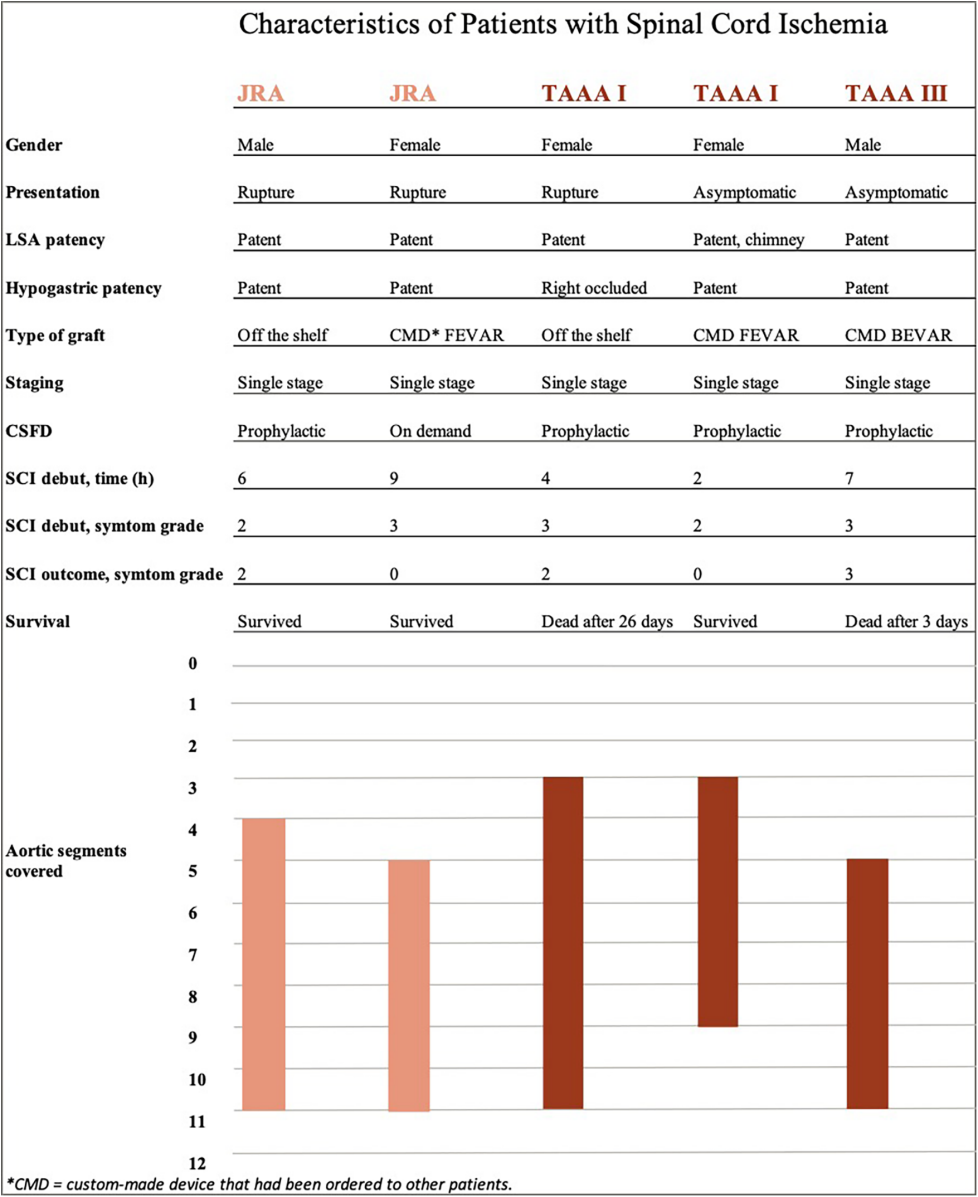
Individual characteristics of patients experiencing spinal cord ischemia. The five patients developing spinal cord ischemia are listed on the x-axis. Individual details regarding spinal cord ischemia are listed below, followed by bars that represent the extent of aortic coverage according to included sealing zones (y-axis).

In total, five patients (2.4%) developed SCI within 30 days postoperative, two after repair of JRA (1.3%, both ruptured) and three after TAAA (6.0%, one ruptured). Four patients who experienced SCI had prophylactical CSFD. After lowering the passive drainage pressure from 10 to 5 mm Hg, one patient had complete symptom regression (grade 3 to 0) while one had partial regress (grade 3–2) and was still not able to stand without assistance until death 26 days postoperatively. Two patients had no regress of symptoms (remained as grade 3 and grade 2, respectively), one of which died three days postoperatively and the other one was discharged with paraplegia. The fifth patient presenting with a ruptured JRA and receiving a FEVAR, had CSFD placed on demand postoperatively, upon which the symptoms regressed completely (grade 3 to 0). Individual details of the patients developing SCI are presented in Figure 1. Patients developing postoperative SCI were more commonly female (3 of 5, *p* = .016), had a preoperative eGFR <60 ml/min/1.73 m^2^ (5 of 5, *p* < .001) and presented with rupture (3 of 5, *p *< .001). SCI developed between 2 and 9 h after the operation, hence all while the protocol was being used. During the duration of this protocol, the patients with SCI had significantly lower minimal MAP than the ones not developing this complication (60 (45–72) mm Hg vs. 73 (68–80) mm Hg, *p* = .015). There were also significant differences between lowest MAP and lowest Hb for ruptured and intact aneurysm after exclusion of the aneurysm sac (MAP 68 (52–70) vs. 75 (68–82) mm Hg, *p *= .002 and Hb 93 (81–107) vs. 102 (93–113) g/L, *p *= .068). No regression analysis was done due to the limited number of events (SCI) in relation to the study population size. The two patients with SCI who died within 30 days had also other major complications, one of them bowel ischemia and renal failure and the other one infection and pulmonary oedema. Those who survived experienced isolated SCI without other complications.

## 4 Discussion

This study confirms that the implementation of the SCI preventive protocol is successful, leading to a sustained decrease of SCI incidence to 2.4% in the total population. This had already been suggested by the initial introduction of the protective protocol, where SCI after TAAA repair in the same tertiary center had decreased and the recovery rates improved (13). Aortic rupture at presentation, female gender, low postoperative MAP and preoperative renal decline were more commonly reported among patients developing SCI, which has been previously documented (5). The presence of rupture may have had several implications that conditioned the development of SCI. This was reinforced by the fact that the two juxtarenal and one of the three TAAA developing SCI presented with ruptured aneurysms. Initially, it is desirable to keep the blood pressure as low as tolerable, to minimize blood loss. Nevertheless, as soon as the aneurysm is excluded, MAP is increased to ensure adequate spinal cord blood. As indicated in the results, however, it is difficult to maintain adequate MAP levels for these patients. The SCI group had even lower MAP than the rupture group as a whole. This reinforces the preventive role of the perfusion pressure as expressed by the MAP and is in line with the evidence of the potential deleterious effect of a too aggressive permissive hypotension in ruptured aneurysms (11, 19). Lastly, rupture may imply more extensive non-staged aortic coverage giving the acute setting with the use of non-custom-made stentgrafts (20). The location of an aneurysm and extent of aortic coverage have previously been described as risk factors (4, 5, 11). Our findings are in line with this since the incidence of SCI after treating TAAA was 6.0% and only 1.3% after JRA. However, there are some important potential confounders; the significantly larger aneurysm diameter, longer operation time and greater bleeding in the TAAA group may all have added to the difference. Furthermore, some attention needs to be drawn to the primary technical success that was slightly lower than the commonly reported. This may also have been driven by the inclusion of urgent patients where compromises are usually done particularly for the target vessels which were the driver of the initial failures. This could in some instances be corrected by reinterventions as evidenced by the secondary success but needs further analysis. Another factor that needs further assessment is the importance of gender. Among the 38 female patients, four presented with rupture and three developed SCI, which was significantly worse than in males. This has previously been suggested by Witheford et al. (21), and better tools to assist in patient selection and preoperative optimization are greatly needed.

The current SCI preventive protocol integrates not only measures to prevent the development of SCI. It also allows its early diagnosis due to hourly neurological wake-up tests and thereby enable the introduction of therapeutic measures that enhance the recovery. The duration of the controls has, nevertheless, been shortened to 12 h in JRA repairs and 36 in TAAA, if patients remain asymptomatic. This strategy seems to be effective considering the timing of the symptom development in the five cases of SCI. However, it presupposes active management of the oxygen delivery to the spinal cord by maintaining the Hb, MAP and saturation levels at the vascular surgery ward post ICU. A further shortening of the duration of the protocol may be difficult to implement considering the limited resources used and the good results obtained. Some other factors may have had a significant contribution to the good results concerning SCI development, especially in patients with TAAA. The staging of the procedures seems to contribute to a significant decrease in the SCI incidence, which was reinforced by the fact that none of the 18 patients with staged procedures developed SCI. However, there is a risk of intercurrent rupture, and some patients may not come to the second stage.

The therapeutic use of the CSFD seems to continue to be an effective tool considering that three out of the five patients experienced symptom regression, two complete and one partial. Furthermore, it is possible that a great number of SCI were prevented as a result of reduced intraspinal pressure due to prophylactic CSFD, as previously indicated by Khan et al. (22). However, the evidence has suggested that CSFD is associated with risks, which was unfortunately confirmed in two patients who suffered subdural hematoma (23, 24). Due to this non negligible complication in addition to decreasing incidence of SCI and the ability of early diagnosis through neurological controls, the usage of CSFD was re-evaluated during the study period. It resulted in a strategy of selective placement of CSFD upon the development of symptoms, or prophylactically in case of very extensive repairs that could not be staged. This approach is also becoming more common in the reports (11, 12, 25–27). Nevertheless, the postoperative placement is also potentially associated with increased risks of complications due to the anticoagulation administrated before the surgery. None of the patients receiving CSFD postoperatively had complications derived from the placement, but in four instances the neurological symptoms could later be attributed to other causes. This underlines the difficulties in the diagnosis and the importance of having dedicated and experienced neurological assessments. Conclusively, further studies are needed to clarify further the role of CSFD in complex EVAR, such as the on-going randomized trial in the USA (28).

Besides the measures taken to diagnose and prevent SCI, some attention needs to be taken into the preoperative risks of developing this complication. Previous reports suggest that preoperative hypertension, poor renal function, COPD, and advanced age are risk factors for developing SCI, which was consistent with the results of this study (5, 29). Some of this risk factors are not modifiable, or the effects of interventions, despite being very impactful, take time such as smoke cessation. Consequently, efforts should also focus on all measures to prevent complications such as postoperative acute kidney damage, particularly by avoiding contrast-induced kidney damage. The potential interplay of the different complications is reinforced by the two patients with SCI who died within 30 days postoperative that also had suffered other major complications. Moreover, the potential serious consequences of the postoperative complications also underline the importance of rescuing the relatively high number of postoperative complications in these patients (30). Regarding the collateral vascular supply to the spinal cord, there was no association between hypogastric or LSA occlusion and SCI development in our study, which has previously been seen (7, 11). However, this may have been influenced by the efforts of preserving these collateral beds and concurs with the findings by Spanos et al. (5) that suggest that CSFD and staging the procedure, both of which has been widely used in this study, minimize the impact of these risk factors.

There are some limitations to this study that need to be addressed. First of all, the individual effects of the measures included in the SCI preventive protocol are difficult to assess, considering they were introduced simultaneously. Furthermore, the usage of CSFD and MEP was altered during the study period and information regarding when and why it was changed is non-available. Secondly, the length of aortic coverage was approximated using the sealing zones, although it would be more accurate with exact measurements. Even more interesting would be to analyze the amount of covered segmental arteries when assessing risk factors of SCI. There is currently an ongoing randomized controlled multicenter trial investigating the safety and efficacy of performing minimally invasive segmental artery coil embolization prior to the EVAR (31). The aim is similar to staging the procedure in abdominal and thoracic sessions, as was partly done in this study, serving to enable the formation of a collateral network to the spinal cord (10). A third limitation of this study would be that some of the variables are interdependent and there is a risk of confounding bias when analyzing the results. However, the total population was not sufficiently large to allow a meaningful regression analysis that would compensate for this. Hence, there is a possible risk of statistical error I or II when comparing the five patients with SCI and the remaining population. However, the limited amount of SCI cases is highly successful and should be viewed as a result of an effective SCI preventive protocol.

### 4.1 Conclusion

During a 6-year period with the routine use of a dedicated SCI preventive protocol, the incidence of SCI after undergoing EVAR of complex aneurysms was 2.4%. Female gender, ruptured aneurysm and low postoperative MAP were non-independently associated with patients developing SCI and their relative contributions could not be assessed. This study also indicates that a standardized SCI preventive protocol is effective in minimizing the development of SCI and allowing for a significant regression of the symptoms. This despite that in patients undergoing elective repair of JRA, the protocol duration was significantly shorter than for TAAA, usually overnight. Further studies are needed to stratify the risk of developing SCI and customize the SCI preventive protocol accordingly.

## Data Availability

The raw data supporting the conclusions of this article will be made available by the authors, without undue reservation.

## References

[1] Greenhalgh RM Brown LC Powell JT Thompson SG Epstein D Sculpher MJ. Endovascular versus open repair of abdominal aortic aneurysm. N Engl J Med. (2010) 362(20):1863–71. 10.1056/NEJMoa0909305 20382983

[2] Oderich GS Tenorio ER Mendes BC Lima GBB Marcondes GB Saqib N Midterm outcomes of a prospective, nonrandomized study to evaluate endovascular repair of Complex aortic aneurysms using fenestrated-branched endografts. Ann Surg. (2021) 274(3):491–9. 10.1097/SLA.0000000000004982 34132698

[3] Lella SK Waller HD Pendleton A Latz CA Boitano LT Dua A. A systematic review of spinal cord ischemia prevention and management after open and endovascular aortic repair. J Vasc Surg. (2022) 75(3):1091–106. 10.1016/j.jvs.2021.10.039 34740806

[4] Aucoin VJ Motyl CM Novak Z Eagleton MJ Farber MA Gasper W Predictors and outcomes of spinal cord injury following complex branched/fenestrated endovascular aortic repair in the US aortic research consortium. J Vasc Surg. (2023) 77(6):1578–87. 10.1016/j.jvs.2023.01.205 37059239

[5] Spanos K Kolbel T Kubitz JC Wipper S Konstantinou N Heidemann F Risk of spinal cord ischemia after fenestrated or branched endovascular repair of complex aortic aneurysms. J Vasc Surg. (2019) 69(2):357–66. 10.1016/j.jvs.2018.05.216 30385148

[6] Katsargyris A Oikonomou K Kouvelos G Renner H Ritter W Verhoeven EL. Spinal cord ischemia after endovascular repair of thoracoabdominal aortic aneurysms with fenestrated and branched stent grafts. J Vasc Surg. (2015) 62(6):1450–6. 10.1016/j.jvs.2015.07.066 26365661

[7] Eagleton MJ Shah S Petkosevek D Mastracci TM Greenberg RK. Hypogastric and subclavian artery patency affects onset and recovery of spinal cord ischemia associated with aortic endografting. J Vasc Surg. (2014) 59(1):89–94. 10.1016/j.jvs.2013.07.007 24188715

[8] Acher C Acher CW Marks E Wynn M. Intraoperative neuroprotective interventions prevent spinal cord ischemia and injury in thoracic endovascular aortic repair. J Vasc Surg. (2016) 63(6):1458–65. 10.1016/j.jvs.2015.12.062 26968081

[9] Weigang E Parker JA Czerny M Lonn L Bonser RS Carrel TP Should intentional endovascular stent-graft coverage of the left subclavian artery be preceded by prophylactic revascularisation? Eur J Cardiothorac Surg. (2011) 40(4):858–68. 10.1016/j.ejcts.2011.01.046 21376612

[10] Heidemann F Tsilimparis N Rohlffs F Debus ES Larena-Avellaneda A Wipper S Staged procedures for prevention of spinal cord ischemia in endovascular aortic surgery. Gefasschirurgie. (2018) 23(Suppl 2):39–45. 10.1007/s00772-018-0410-z 30147243 PMC6096720

[11] Marcondes GB Cirillo-Penn NC Tenorio ER Adam DJ Timaran C Austermann MJ Multicenter study to evaluate endovascular repair of extent I–III thoracoabdominal aneurysms without prophylactic cerebrospinal fluid drainage. Ann Surg. (2023) 278(2):e396–404. 10.1097/SLA.0000000000005653 35925761

[12] Aucoin VJ Eagleton MJ Farber MA Oderich GS Schanzer A Timaran CH Spinal cord protection practices used during endovascular repair of complex aortic aneurysms by the U. S. aortic research consortium. J Vasc Surg. (2021) 73(1):323–30. 10.1016/j.jvs.2020.07.107 32882346

[13] Dias NV Sonesson B Kristmundsson T Holm H Resch T. Short-term outcome of spinal cord ischemia after endovascular repair of thoracoabdominal aortic aneurysms. Eur J Vasc Endovasc Surg. (2015) 49(4):403–9. 10.1016/j.ejvs.2014.12.034 25680656

[14] Chaikof EL Blankensteijn JD Harris PL White GH Zarins CK Bernhard VM Reporting standards for endovascular aortic aneurysm repair. J Vasc Surg. (2002) 35(5):1048–60. 10.1067/mva.2002.123763 12021727

[15] Fillinger MF Greenberg RK McKinsey JF Chaikof EL. Society for vascular surgery ad hoc committee on TRS. Reporting standards for thoracic endovascular aortic repair (TEVAR). J Vasc Surg. (2010) 52(4):1022–33.e15. 10.1016/j.jvs.2010.07.008 20888533

[16] Oderich GS Forbes TL Chaer R Davies MG Lindsay TF Mastracci T Reporting standards for endovascular aortic repair of aneurysms involving the renal-mesenteric arteries. J Vasc Surg. (2021) 73(1S):4S–52. 10.1016/j.jvs.2020.06.011 32615285

[17] Tsilimparis N Fiorucci B Debus ES Rohlffs F Kölbel T. Technical aspects of implanting the t-branch off-the-shelf multibranched stent-graft for thoracoabdominal aneurysms. J Endovasc Ther. (2017) 24(3):397–404. 10.1177/1526602817690730 28164732

[18] Resch TA Dias NV Sobocinski J Sonesson B Roeder B Haulon S. Development of off-the-shelf stent grafts for juxtarenal abdominal aortic aneurysms. Eur J Vasc Endovasc Surg. (2012) 43(6):655–60. 10.1016/j.ejvs.2012.01.022 22342691

[19] Powell JT Sweeting MJ Thompson MM Ashleigh R Bell R Gomes M Endovascular or open repair strategy for ruptured abdominal aortic aneurysm: 30 day outcomes from IMPROVE randomised trial. Br Med J. (2014) 348(f7661). 10.1136/bmj.f7661 24418950

[20] Spath P Tsilimparis N Furlan F Hamwi T Prendes CF Stana J. Additional aortic coverage with an off the shelf, multibranched endograft compared with custom made devices for endovascular repair of pararenal abdominal aortic aneurysms. Eur J Vasc Endovasc Surg. (2023) 65(5):710–8. 10.1016/j.ejvs.2023.01.030 36707021

[21] Witheford M Chong DST Martin-Gonzalez T Van Calster K Davis M Prent A Women undergoing endovascular thoracoabdominal aortic aneurysm repair differ significantly from their male counterparts preoperatively and postoperatively. J Vasc Surg. (2020) 71(3):748–57. 10.1016/j.jvs.2019.05.053 31477478

[22] Khan NR Smalley Z Nesvick CL Lee SL Michael LM 2nd, The use of lumbar drains in preventing spinal cord injury following thoracoabdominal aortic aneurysm repair: an updated systematic review and meta-analysis. J Neurosurg Spine. (2016) 25(3):383–93. 10.3171/2016.1.SPINE151199 27058497

[23] Wynn MM Mell MW Tefera G Hoch JR Acher CW. Complications of spinal fluid drainage in thoracoabdominal aortic aneurysm repair: a report of 486 patients treated from 1987 to 2008. J Vasc Surg. (2009) 49(1):29–34. 10.1016/j.jvs.2008.07.076 18951749

[24] Borghese O Brisard L Le Corvec T Hauguel A Guimbretière G Maurel B. Spinal cord protection during thoracic and thoracoabdominal endovascular aortic repair: 5-year results of a preventive protocol including prophylactic cerebrospinal fluid drainage in high-risk patients. J Endovasc Ther. (2023) 15266028231215972. 10.1177/15266028231215972 38084383

[25] Riambau V Bockler D Brunkwall J Cao P Chiesa R Coppi G Editor’s choice—management of descending thoracic aorta diseases: clinical practice guidelines of the European society for vascular surgery (ESVS). Eur J Vasc Endovasc Surg. (2017) 53(1):4–52. 10.1016/j.ejvs.2016.06.005 28081802

[26] Zhang Z Zhou Y Lin S Xiao J Ai W Zhang WW. Systematic review and meta-analysis of association of prophylactic cerebrospinal fluid drainage in preventing spinal cord ischemia after thoracic endovascular aortic repair. J Vasc Surg. (2022) 75(4):1478–89.e5. 10.1016/j.jvs.2021.10.050 34793925

[27] Babocs D Dias-Neto M Vacirca A Huang Y Baghbani-Oskouei A Jakimowicz T Outcomes of elective and non-elective fenestrated-branched endovascular aortic repair for treatment of thoracoabdominal aortic aneurysms. J Vasc Surg. (2024) 79(4):568–77. 10.1016/j.jvs.2024.01.156 37395613

[28] Blakeslee-Carter J Novak Z Jansen JO Schanzer A Eagleton MJ Farber MA Prospective randomized pilot trial comparing prophylactic vs therapeutic cerebrospinal fluid drainage during complex endovascular thoracoabdominal aortic aneurysm repair. J Vasc Surg. (2024) 80(1):11–9. 10.1016/j.jvs.2024.02.041 38614137

[29] Awad H Ramadan ME El Sayed HF Tolpin DA Tili E Collard CD. Spinal cord injury after thoracic endovascular aortic aneurysm repair. Can J Anaesth. (2017) 64(12):1218–35. 10.1007/s12630-017-0974-1 29019146 PMC5954412

[30] D'Oria M Scali S Mao J Szeberin Z Thomson I Beiles B Association between hospital volume and failure to rescue after open or endovascular repair of intact abdominal aortic aneurysms in the VASCUNET and international consortium of vascular registries. Ann Surg. (2021) 274(5):e452–9. 10.1097/SLA.0000000000005044 34225297

[31] Petroff D Czerny M Kolbel T Melissano G Lonn L Haunschild J Paraplegia prevention in aortic aneurysm repair by thoracoabdominal staging with ‘minimally invasive staged segmental artery coil embolisation’ (MIS(2)ACE): trial protocol for a randomised controlled multicentre trial. BMJ Open. (2019) 9(3):e025488. 10.1136/bmjopen-2018-025488 30837256 PMC6429943

