# Supplementary material for: A dedicated preventive protocol sustainably avoids spinal cord ischemia after endovascular aortic repair
Source: Front Cardiovasc Med. 2024 Aug 1;11:1440674. doi: 10.3389/fcvm.2024.1440674 (PMC11324596; doi:10.3389/fcvm.2024.1440674)
Supplement: Supplementary file 1 [file Datasheet1.docx]

Supplementary Material

## Supplementary Figures
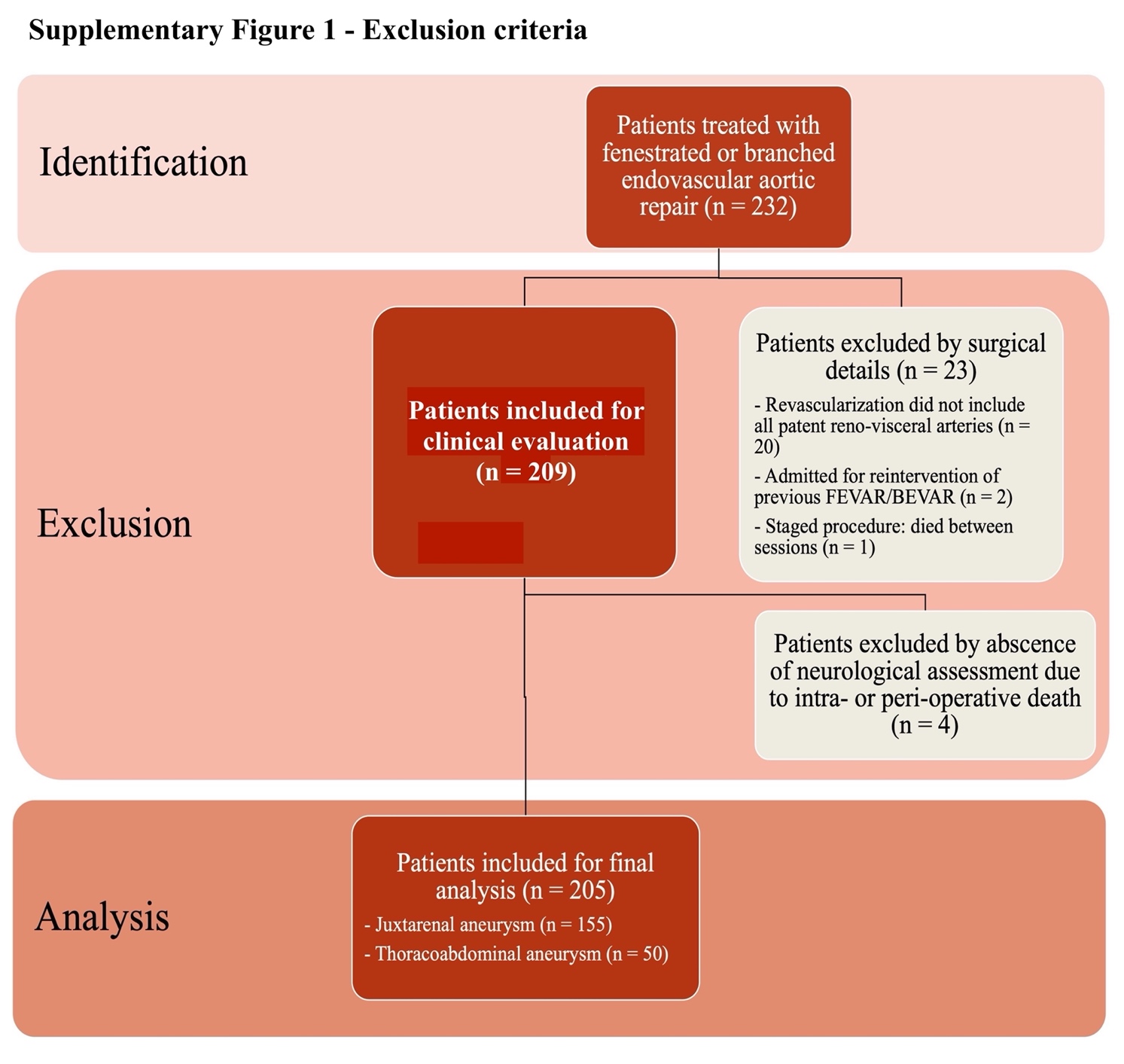
Supplementary Figure 1. Patient inclusion to the final analysis.

**Supplementary Figure 2.** Depiction of the extent of the aortic coverage according to the sealing zones and divided by the preoperative aneurysm extent.
